# Quality of life in persons with dementia using regional dementia care network services in Germany: a one-year follow-up study

**DOI:** 10.1186/s12955-018-0990-z

**Published:** 2018-09-14

**Authors:** Johannes Gräske, Annika Schmidt, Sylvia Schmidt, Franziska Laporte Uribe, Jochen René Thyrian, Bernhard Michalowsky, Susanne Schäfer-Walkmann, Karin Wolf-Ostermann

**Affiliations:** 1 HTW des Saarlandes, Faculty of Social Sciences, Department of Health and Nursing, Goebenstr. 40, 66117 Saarbrücken, Germany; 2 Institute for Public Health and Nursing Research (IPP), University of Bremen, Grazer Str. 4, 28359 Bremen, Germany; 3 Competence Centre for Clinical Studies, University of Bremen, Linzer Str. 4, 28359 Bremen, Germany; 4 German Centre for Neurodegenerative Diseases (DZNE) site Witten, Stockumer Str. 12, 58453 Witten, Germany; 5 German Centre for Neurodegenerative Diseases (DZNE), site Rostock / Greifswald, Ellernholzstr. 1-2, 17489 Greifswald, Germany; 6 Institute of Applied Social Sciences (IfaS) at DHBW Stuttgart, Rotebühlstraße 131, 70197 Stuttgart, Germany; 7 Institute for Community Medicine, Department of Epidemiology of Health Care and Community Health, University Medicine, Greifswald, Germany

**Keywords:** Dementia, Regional dementia care networks, Quality of life, Primary dementia care

## Abstract

**Background:**

The majority of individuals with dementia live in the community; thus, regional dementia care networks are becoming increasingly more important for the provision of care. To date, four different types of dementia care networks have been identified in Germany (stakeholder, organisation, hybrid, mission); however, the effect on the quality of life of persons with dementia using such network services has not yet been examined. Moreover, the possible differences in the effect on the quality of life among the four types of dementia care networks have not been investigated. Therefore, the aim of the present study was to describe the changes over time in the quality of life of persons with dementia, assessing the association with the different types of dementia care networks.

**Methods:**

Within the DemNet-D study, face-to-face interviews with persons with dementia and their primary caregivers were conducted to collect data of typical outcome parameters, such as quality of life (Quality of Life Alzheimers Disease: QoL-AD), sociodemographic data, social index (Scheuch−Winkler), depression (Geriatric Depression Scale: GDS), challenging behaviour (Cohen−Mansfield Agitation Inventory: CMAI), capacities of daily living (Instrumental Activity of Daily Living: IADL), impairment due to dementia (FAST), and caregiver burden. In addition to these parameters, the differences in quality of life scores among the four types of dementia care networks were analysed using multi-level analysis.

**Results:**

In total, 407 persons with dementia (79.1 years; 60.1% female) and their caregivers were included in the analysis. Over 75% of the persons with dementia showed moderate to (very) severe impairments of dementia and at least one challenging behaviour. At baseline, 60.6% had a low social index. Quality of life was stable over one-year on a level slightly above average (baseline 29.1; follow-up 28.7). Multi-level analyses (*p* <  0.001; R^2^ = 0.183) show that persons with dementia with higher QoL-AD scores at baseline were associated with a decline at follow-up. No significant differences among the types of dementia care networks were found.

**Conclusion:**

Users of dementia care network services showed a stable QoL-AD score over time at a level slightly above average, indicating no decrease or worsening over time as expected. Therefore, dementia care network services can be considered as a beneficial model of care in terms of the quality of life of persons with dementia, regardless of their special organisational type.

## Background

With the increasing number of older people, the number of persons worldwide with dementia is expected to rise to 115 million by 2050 [1], and in Germany, this increase is estimated to reach approximately 1.6–3.0 million [2]. Dementia comprises different forms; the most prevalent form is Alzheimer’s disease, which remains incurable. It is characterised by a decline in the cognitive functions of affected persons [3]. Prevalence and incidence studies have shown the high impact of dementia on current healthcare systems [4]. Informal caregivers predominantly cover the support of persons with dementia [5]; however, informal caregiving causes stress and psychological distress [6]. These facts raise questions regarding the future adequate provision of care for persons with dementia. Primary care is most favourable from the perspective of older adults [7], but is considered challenging for healthcare providers. The necessary cooperation with other healthcare providers often causes delays or lack of information about the condition of persons with dementia, complicating the provision of adequate healthcare. To evaluate interventions in dementia care, the quality of life of persons with dementia is seen as an appropriate indicator [8].

### Quality of life of persons with dementia

Due to the lack of curative treatments, quality of life is seen as an adequate parameter for the evaluation of dementia care [9], and is associated with the individual satisfaction with key areas of life [10]. There is no generally accepted definition of quality of life of persons with dementia, and it is regarded as a broad multidimensional concept [11] including subjective (e.g. comfort), objective (e.g. neglect), and personal (e.g. need for care) needs [12]. Therefore, quality of life indicates effective care for persons with dementia in more aspects than merely changes in biomarkers [13]. Quality of life ratings are possible as self- and proxy-ratings; and for the best agreement with self-ratings, proxy-rated quality of life should be carried out by primary (informal) caregivers [14]. Proxy-rated quality of life of persons with dementia, among others, depends on factors associated with the level of caregiver burden [15, 16]. With respect to sociodemographic characteristics (such as age and gender) and outcome parameters (such as depression and challenging behaviour), there exists inconsistent evidence with a slight tendency towards an association with quality of life [17]. In general, the quality of life of persons with dementia declines with the progression of the disease. Indeed, during a one-year follow-up, Vogel et al., [18] identified a significant decline in the quality of life of community-dwelling persons with dementia of 2.0 points, as measured using the Quality of Life Alzheimer’s Disease (QoL-AD) scale.

### Dementia care

It is common to provide individualised tailored care meeting the above-mentioned needs of persons with dementia to improve their quality of life, or at least to maintain it in a stable state; however, care provision for persons with dementia is seen as complex and challenging [19]. Often, healthcare provision cannot be guaranteed in an adequate manner due to the lack of general practitioners and specialists, especially in rural areas [20]. Additionally, the German healthcare system is fragmented (e.g. out- and inpatient services) with interface problems among different sectors, causing lack of treatment and inadequate care. Therefore, in Germany, regional dementia care networks were set up to overcome this gap, providing a coordinated and structured care approach.

### Regional dementia care networks

Dementia care networks are regarded as a model of integrated collaborative care [21], defined by the World Health Organisation as ‘the management and delivery of health services so that clients receive a continuum of preventive and curative services, according to their needs over time and across different levels of the health system’ [22]. To achieve this aim, dementia care networks formalise multi-professional collaboration of healthcare providers (e.g. general practitioners, specialists, nurses, therapists, as well as local authorities).

### Regional dementia care networks in Germany

In recent years, regional dementia care networks have been established in Germany. Such networks can be explained as a legitimatised societal approach to link different dementia support services and stakeholders. These dementia care networks aim to address the high degree of fragmentation that characterises the German healthcare system [23]; however, there is no common definition that fits the heterogeneity and current innovative dynamics of dementia care networks. They vary widely in different aspects including size, number of stakeholders and staff, as well as funding and cooperation structures [24]. In general, dementia care networks often use innovative measures to meet the specific needs of persons with dementia in their region. For such complex care arrangements as those needed in dementia care, a welfare-mix is characteristic; there are market elements and stakeholders of the Third Sector, as well as public organisations and private engagement (e. g. private caregivers and peer-support groups). Most dementia care networks commonly aim to provide direct, appropriate, tailored, and timely care for persons with dementia [25, 26]. Other dementia care networks provide information for persons with dementia and their relatives, society, and federal and local institutions (e.g. police departments and pharmacies) [27]. They intend to maintain or increase the quality of life of persons affected by the disease, as well as to reduce caregiver burden. A more differentiated description of dementia care networks has been investigated as part of the DemNet-D study but was not the focus of the current analysis.

In summary, services provided by dementia care networks can include geriatric assessments and treatment, as well as sharing information and coordinating services. During the first step of the German dementia care network study (DemNet-D), four different governance types of dementia care networks [23] were identified:

- Stakeholder: Dementia care networks of this type focus on providing support by identifying and linking regional care providers and actively involving stakeholders. They refer their work to the (social) environment, and the specific involvement of external care providers gives the opportunity to improve achievement of the objectives. The aim of stakeholder dementia care networks is predominantly to educate persons with dementia and their family members.
- Organisation: This type of dementia care network is characterised by a high level of internal formal governance and a defined control centre. Creating and following formal and informal structures, dementia care network stakeholders intend to achieve a high level of efficacy and effectiveness of support structures provided by the network. Through this highly defined governance structure, achievement of the objectives is independent from the individuals. The main network aims focus more on intrinsic structures rather than on external interventions.
- Hybrid: Dementia care networks of this type are characterised by the ability to quickly adapt their strategies to accommodate changing requirements regarding environmental or legal regulations. This feature allows these dementia care networks to act on a state-of-the-art level, and therefore to play an active part in the provision of healthcare services. The level of involvement of network stakeholders depends on their goals as a stakeholder.
- Mission: This type of dementia care network is characterised by user-focused, specific care-related aims that are followed through consistently. All resources and features aim to achieve care-related objectives. To realise these objectives, mission-related dementia care networks must know in detail the needs of persons with dementia as well as care providers in their region.

The previously described characteristics of each governance type are predominantly typical of a certain type of dementia care network. Additional characteristics may rarely occur.

In a previous study, the evaluation of one dementia care network of the stakeholder type in Germany showed a stable quality of life score during a one-year follow-up [28]. However, these results are not easily generalisable and there is a lack of evaluation of a higher number of different and diverse dementia care networks. Therefore, the present paper aimed to explore the changes in the quality of life of persons with dementia using dementia care network services over a period of 1 yr. Additionally, a deeper analysis was conducted to show differences among the use of four different types of dementia care networks.

## Methods

The present paper was based on data from the multi-centre, interdisciplinary evaluation study of dementia care networks in Germany, DemNet-D, which was conducted as a one-year observational quasi-experimental study (2012–2015). In total, 13 dementia care networks from all over Germany participated in the present study. Due to practical reasons, no control group defined as persons not part of a dementia care network was included, since most persons with dementia or their family members are not necessarily aware of whether their healthcare provider is part of a dementia care network. Therefore we could not ensure that participants of a control group would not have used services provided by dementia care networks.

### Sample

The DemNet-D study included persons with dementia and their primary caregivers when they used the services of 1 of the 13 participating dementia care networks. Detailed inclusion criteria for persons with dementia were:

- utilising one of the participating dementia care networks;
- having a formal diagnosis of dementia;
- living at home;
- having a primary caregiver (e.g. relative, friend, or nurse);
- giving written informed consent (for persons with dementia with a legal representative, written informed consent was obtained from the representative).

A primary caregiver was defined as a person with primary responsibility for a person with dementia; who could be an informal caregiver (a family member/friend/neighbour without professional training as a caregiver and who did not receive payment for his/her support other than a constant attendance allowance) or a professional caregiver. The focus of the present study was on informal caregivers, but professional caregivers were not excluded since dementia care networks and their structures have not been previously described in a larger study in Germany [29]. Participants were excluded from the analysis if they withdrew their informed consent, moved outside the region of the participating dementia care network, or died.

### Data collection

Due to German data protection regulations, dementia care networks are not allowed to forward contact details of possible participants without informed consent; therefore, employees of the dementia care networks recruited the study participants. Primary data were collected by specially trained nurses using face-to-face interviews with persons with dementia and their informal caregivers conducted at home. Data collection included sociodemographics (age, gender, and living situation), and the social index was measured using the Scheuch−Winkler Index [30], which allows the categorisation of persons with dementia into three social classes (low, middle, and high).

#### Quality of life – Quality of life Alzheimer’s disease

The quality of life of persons with dementia was the main outcome parameter of the present study, and was measured using the proxy-rated Quality of Life Alzheimer’s Disease (QoL-AD) scale [31]. The QoL-AD consists of 13 items (e.g. family and mood), which could be rated from *rare* (1 point) to *often* (4 points). The results are presented as a sum score (theoretical range: 13–52), with higher scores indicating a better quality of life. The German version shows good psychometric properties [32, 33].

#### Impairment by dementia – Functional assessment staging

Using Functional Assessment Staging (FAST), the informal caregiver rated 18 items (e.g. ability to do complex tasks) regarding the impairment due to dementia. In total, there are seven stages (1 = *no impairment* up to 7 = *very severe impairment*) [34]. Due to the informal caregiver rating, ratings by persons with a medical background (e.g. nurse or general practitioner) may show different results.

#### Challenging behaviour – Cohen−Mansfield agitation inventory

The proxy-rated Cohen−Mansfield Agitation Inventory (CMAI) [35] was used to evaluate the challenging behaviour of persons with dementia. The informal caregiver rated 29 behaviours that are associated with challenging behaviour (e.g. kicking and biting) on a seven-point Likert scale (*never* to *several times an hour*). The analysis indicates the presence of agitated, aggressive, and physically non-aggressive behaviours. In addition, the presence of at least one of these behaviours was evaluated.

#### Depression – Geriatric depression scale

The self-rating instrument, Geriatric Depression Scale (GDS) [36], contains 15 items. The dichotomous (*yes*/*no*) answers are summed (theoretical range: 0*–*15), with higher scores indicating a higher level of depression. The German version shows good psychometric properties [37].

#### Activities of daily living – Instrumental activities of daily living

Using the Instrumental Activities of Daily Living scale (IADL) [38], the informal caregiver rated eight capacities of daily living. The total score ranges from 0 to 8, with higher scores indicating a higher independence. The German version shows good psychometric properties [39].

#### Caregiver burden – Berliner Inventar zur Angehörigenbelastung – Demenz

The “Berliner Inventar zur Angehörigenbelastung – Demenz” [Berlin Inventory of Burden of Family Members – Dementia] (BIZA-D) [40] measures 20 aspects of caregiver burden (e.g. subjective burden due to changing behaviours or burden due to practical care tasks). Due to the strong association between capacities of daily living and caregiver burden [41], only the category “burden due to practical care tasks” was used for in-depth analysis in the present paper. This category consists of six subscales including 25 items, which are ranked on a five-point Likert scale in terms of how strongly every task is perceived as a burden. A higher total score (theoretical range: 0–16) indicates a higher level of burden. An in-depth description, including psychometric properties, has been published elsewhere [42].

#### Data analysis

Data description was conducted with typical descriptive values (mean and standard deviation) and associations between two variables were analysed using a *t*-test and Chi-square test. The main outcome was the difference in quality of life after 1 yr (follow-up after 1 yr minus the baseline value) measured using the QoL-AD. The influence of the type of dementia care network, as well as further factors on the change in the quality of life over 1 yr, was analysed by multivariate linear mixed models, using maximum-likelihood estimation. Dementia care networks were included as random effects with the rationale that people within the same network have similar characteristics.

This model included typical parameters, which are described as being associated with proxy-rated quality of life, as fixed effects [17]: gender (male or female); living situation (living together, nearby, or at a greater distance); social index (Scheuch–Winkler: low, middle, or high class); presence of at least one challenging behaviour (yes or no); the four types of dementia care networks; age (in years); depression (GDS); capacities of daily living (IADL); caregiver burden (BIZA-D); and quality of life at baseline (QoL-AD). Since the difference between follow-up and baseline was considered, only those participants who had observed values at both time points were included in the analysis. Losses to follow-up were excluded. No imputations were performed, leading to the exclusion of observations with missing values in the fixed effects. The statistical significance was set to 5%, with all analyses conducted following the evaluation of model assumptions using SPSS® (v22) and R 3.1.2.

## Results

In total, 13 dementia care networks (*n* = 8 in urban and *n* = 5 in rural areas) participated in the DemNet-D study. Three dementia care networks were identified as *Stakeholder*, *Organisation*, or *Mission* type, each; and the remaining four networks were identified as *Hybrid* networks. At baseline, *n* = 560 persons with dementia and their informal caregivers were included in the present study.

### Sample at baseline

Sociodemographic characteristics are presented in Table 1. At baseline, persons with dementia were 79.7 years (mean) old and predominantly woman (57.0%). The health-related outcomes regarding dementia show a severe level of impairment by dementia (FAST median: 6) with several consecutive impairments (e.g. IADL mean: 2.0). A total of 75.4% of participating persons with dementia had at least one challenging behaviour (CMAI). An in-depth description of the quality of life of persons with dementia entering the study (baseline results) has been published elsewhere [43, 44].

**Table 1:** Sample characteristics

		total (*n* = 560)			*p*-value	follow-up		*p*-value
			follow-up (*n* = 407)*	loss to follow-up (*n* = 153)		T0 (*n* = 407)*	T1 (*n* = 407)*	
Age in years (mean, SD)		79.7 (8.4)	79.1 (8.5)	81.5 (8.1)	0.003^a^	n/a	n/a	n/a
Sex (n, %)								
Female		319 (57.0)	244 (60.1)	81 (53.6)	0.101^b^	n/a	n/a	n/a
Male		232 (41.4)	162 (39.9)	70 (46.4)		n/a	n/a	
Living situation of caregiver (*n*, %)								
Together		**337 (69.7)**	**252 (62.4)**	**85 (57.8)**	**0.014** ^**c**^	**247 (62.8)**	**226 (57.5)**	**< 0.001** ^**d**^
Nearby		**127 (22.7)**	**99 (24.5)**	**28 (19.0)**		**97 (24.7)**	**105 (26.7)**	
Greater distance		**87 (15.5)**	**53 (13.1)**	**34 (23.1)**		**49 (12.5)**	**62 (15.8)**	
Social Index (Scheuch-Winkler) (n, %)								
Low		248 (60.6)	183 (60.6)	65 (60.7)	0.810^c^	n/a	n/a	
Middle		121 (29.6)	91 (30.1)	30 (28.0)		n/a	n/a	
High		40 (9.8)	28 (9.3)	12 (11.2)		n/a	n/a	
Impairment by dementia (FAST, 0–7) (n, %)								
Up to moderate (FAST ≤5)		42 (7.5)	**35 (8.9)**	**7 (4.8)**	**< 0.016** ^**c**^	**30 (7.8)**	**23 (6.0)**	**< 0.001** ^**d**^
Severe (FAST 6)		229 (40.9)	**177 (44.9)**	**52 (35.6)**		**174 (45.4)**	**136 (35.5)**	
Very severe (FAST 7)		269 (48.0)	**182 (46.2)**	**87 (59.6)**		**179 (46.7)**	**224 (58.5)**	
Depression (GDS, 0–15) (mean, SD)		4.6 (3.4)	4.6 (3.4)	4.6 (3.4)	0.967^a^	**4.4 (3.2)**	**4.6 (3.4)**	**< 0.001** ^**e**^
Challenging behavior (CMAI) (n; %)								
Physically non-aggressive behavior	yes	344 (61.4)	152 (37.9)	52 (35.4)	0.330^b^	**152 (37.9)**	**171 (42.6)**	**< 0.001** ^**f**^
	no	204 (36.4)	249 (62.1)	95 (64.6)		**249 (62.1)**	**230 (57.4)**	
Verbally agitated behavior	yes	330 (58.9)	161 (40.0)	60 (40.3)	0.519^b^	**161 (40.0)**	**155 (38.6)**	**< 0.001** ^**f**^
	no	221 (39.5)	241 (60.0)	89 (59.7)		**214 (60.0)**	**247 (61.4)**	
Aggressive behavior	yes	85 (15.2)	59 (14.8)	26 (17.6)	0.251^b^	**59 (14, 8)**	**60 (15.0)**	**< 0.001** ^**f**^
	no	462 (82.5)	340 (85.2)	122 (82.4)		**340 (85.2)**	**339 (85.0)**	
Mind. 1 challenging behavior	yes	422 (75.4)	307 (76.4)	115 (78.2)	0.369^b^	**307 (76.4)**	**321 (79.1)**	**< 0.001** ^**f**^
	no	127 (22.7)	95 (23.6)	32 (21.8)		**95 (23.6)**	**84 (20.9)**	
Capacities of daily living (IADL, 0–8) (mean, SD)		**2.0 (1.9)**	**2.2 (1.9)**	**1.5 (1.6)**	**< 0.001** ^**a**^	**2.2 (1.9)**	**1.5 (1.7)**	**< 0.001** ^**e**^
Quality of life (QoL-AD, 13–52), (mean, SD)		**28.6 (5.5)**	**29.0 (5.4)**	**27.8 (5.7)**	**0.024** ^**a**^	**29.1 (5.4)**	**28.7 (5.4)**	**< 0.001** ^**e**^
Caregiver Burden (BIZAD ADL, 0–16) (mean, SD)		6.2 (4.9)	6.0 (5.0)	6.7 (4.8)	0.113^a^	n/a	n/a	n/a

^a^t-test; ^b^ Fisher exact test; ^c^ chi-square-test; ^d^ Bowker’s Test; ^e^ paired sample t-test; ^f^ McNemar’s Test; DCN: regional dementia care network, *SD* standard deviation, *FAST* Functional Assessment Staging, *GDS* Geriatric Depression Scale, *CMAI* Cohen-Mansfield-Agitation Inventory, *IADL* Instrumental Activities of Daily Living, QoL-AD Quality of Life Alzheimers Disease, BIZA-D Berliner Inventar zur Angehörigenbelastung – Demenz, n/a not applicable, underlined values are most favorable values, bold indicates significant results at significance level 0.05. * due to missing values, sample size may not sum up to *n* = 407 and therefore values may differ from those of the follow-up sample (column 3)

**Fig 1.**
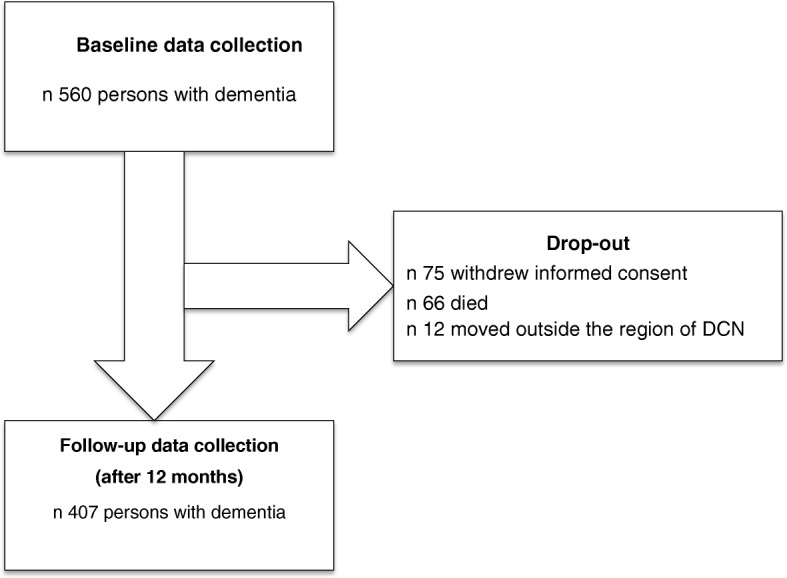
Flowchart

During the one-year observation time, 153 persons with dementia dropped out due to retraction of their informed consent (*n* = 75), death (*n* = 66), or a move outside the region of their dementia care network (*n* = 12) (Fig. 1).

Group differences between persons with a follow-up interview and those who dropped out could be found for age, living situation, capacity of daily living (IADL), and impairment by dementia (FAST). Persons who dropped out were older, their caregivers more often were living in greater distance and had lower capacities of daily living at baseline. The proportion of persons with more severe impairment by dementia (FAST) was also higher. In summary, more strongly affected people dropped out of the study.

With respect to the four different types of dementia care networks, several significant differences were found as shown in Table 2. There was a significant difference (ANOVA: *p* = 0.003) in QoL-AD scores between the stakeholder and mission type (*post-hoc*: *p* <  0.001) dementia care networks, with higher QoL-AD scores for mission networks. The mission dementia care network had lower depression (GDS) scores and was significantly different (ANOVA: *p* <  0.001) from the stakeholder (*post-hoc*: *p* <  0.001) and organisation (*post-hoc*: *p* = 0.001) dementia care networks. Furthermore, the caregiver burden differed significantly (ANOVA: *p* <  0.001) among dementia care networks types, with a lower burden found in users of the mission type as compared with the stakeholder type (*post-hoc*: *p* = 0.010). In addition, there were significantly less male users of the mission dementia care networks, which also had more users with a high social index as compared with the other types of dementia care networks.

**Table 2:** Sample characteristics at baseline and types of DCN

		Stakeholder (*n* = 119)	Organization (*n* = 82)	Hybrid (*n* = 109)	Mission (*n* = 97)	*p*-value
Age in years (mean, sd)		78.7 (8.8)	80.6 (8.0)	78.5 (8.3)	79.1 (8.6)	0.333^**a**^
Sex (n, %)						
female		43 (36.1)	31 (38.3)	40 (36.7)	48 (49.5)	< 0.174^b^
male		76 (63.9)	50 (61.7)	69 (63.3)	49 (50.5)	
Living situation of caregiver (n, %)						
Together		**78 (66.1)**	**50 (62.5)**	**60 (55.0)**	**64 (66.0)**	**0.025** ^**b**^
nearby		**33 (28.0)**	**22 (27.5)**	**27 (24.8)**	**17 (17.5)**	
Greater distance		**7 (5.9)**	**8 (10.0)**	**22 (20.2)**	**16 (16.5)**	
Social Index (Scheuch-Winkler) (n, %)						
low		**65 (67.0)**	**16 (36.4)**	**60 (75.9)**	**42 (51.2)**	**< 0.001** ^**b**^
middle		**27 (27.8)**	**25 (56.8)**	**11 (13.9)**	**28 (34.1)**	
high		**5 (5.2)**	**3 (6.8)**	**8 (10.1)**	**12 (14.6)**	
Impairment by dementia (FAST, 0–7) (n, %)						
up to moderate (FAST ≤5)		8 (7.1)	5 (6.3)	8 (7.4)	14 (14.9)	0.082^b^
severe (FAST 6)		59 (52.2)	32 (40.5)	53 (49.1)	33 (35.1)	
very severe (FAST 7)		46 (40.7)	42 (53.2)	47 (43.5)	47 (50.0)	
Depression (GDS)						
score (0–15); mean (sd)		**5.4 (3.7)**	**5.3 (3.4)**	**4.4 (3.3)**	**3.3 (2.9)**	**< 0.001** ^**a**^
Challenging behavior (CMAI) (n, %)						
Physically non-aggressive behavior	yes	152 (37.9)	52 (35.4)	0.330^b^	249 (62.1)	< 0.101^f^
	no	249 (62.1)	95 (64.6)		158 (38.8)	
Verbally agitated behavior	yes	161 (40.0)	60 (40.3)	0.519^b^	241 (60.0)	< 0.648^f^
	no	241 (60.0)	89 (59.7)		166 (40.0)	
Aggressive behavior	yes	59 (14.8)	26 (17.6)	0.251^b^	59 (14,8)	< 1.000^f^
	no	340 (85.2)	122 (82.4)		348 (85.2)	
At least 1 challenging behavior	yes	307 (76.4)	115 (78.2)	0.369 ^b^	307 (76.4)	< 0.272^f^
	no	95 (23.6)	32 (21.8)		100 (23.6)	
Capacities of daily living (IADL, 0–8); mean (sd)		**1.9 (2.1)**	**1.9 (1.6)**	**2.3 (1.9)**	**2.5 (2.0)**	**< 0.050** ^**a**^
Quality of life (QoL-AD, 13–52); mean (sd)		**27.8 (5.6)**	**28.6 (5.3)**	**29.0 (5.4)**	**29.0 (5.4)**	**0.003** ^**a**^
Caregiver Burden (BIZAD_ADL, 0–16) (mean, sd)		**7.1 (5.1)**	**6.3 (5.1)**	**5.4 (4.5)**	**4.9 (5.0)**	**0.008** ^**a**^

^a^ ANOVA; ^b^ Fisher’s exact test; ^c^ chi-square-test; ^d^ Bowker’s Test; ^e^ paired sample t-test; ^f^ McNemar’s Test, DCN: regional dementia care network, *sd* standard deviation, *FAST* Functional Assessment Staging, *GDS* Geriatric Depression Scale, *CMAI* Cohen-Mansfield-Agitation Inventory, *IADL* Instrumental Activities of Daily Living, *QoL-AD* Quality of Life Alzheimer’s Disease, *BIZA-D* Berliner Inventar zur Angehörigenbelastung – Demenz; underlined values are most favorable values, bold indicates significant results at significance level 0.05

### Changes over time

**Fig 2.**
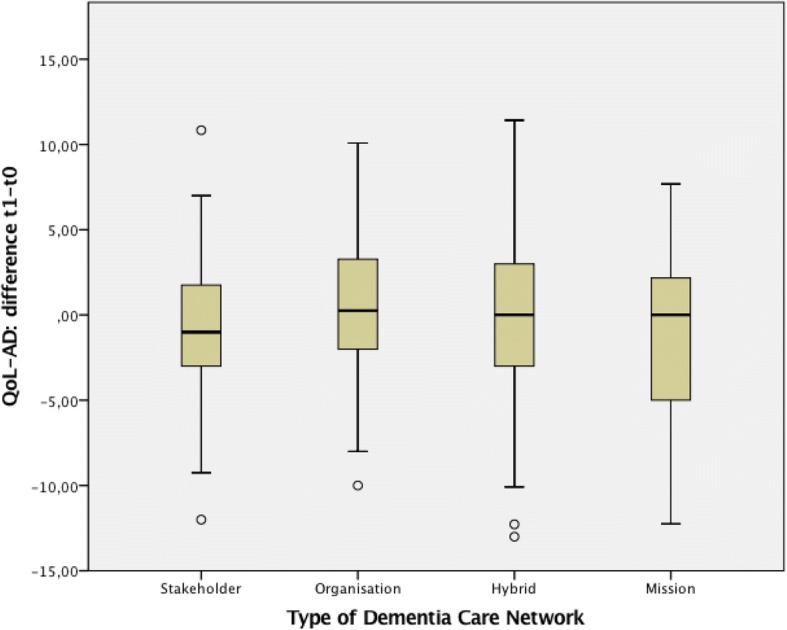
Changes of Quality of Life Scores over one year, by Types of Dementia Care Networks

During the one-year follow-up, the QoL-AD score remained relatively stable over time, with an average decline (follow-up minus baseline score) of 0.4. The descriptive comparison of quality of life changes according to the different types of dementia care networks is displayed in Fig. 2, and no statistically significant differences were observed. Additionally, the sample showed progressive symptoms of dementia (Table 1). Participants were more often living with their caregivers at baseline compared to follow-up (62.8% vs. 57.5%, Bowker *p* <  0.001). The proportion of persons with dementia with very severe impairments due to dementia significantly increased from 46.7 to 58.5% over one-year (Bowker *p* <  0.001). Furthermore, symptoms of depression increased (4.4 to 4.6, paired *t*-test *p* <  0.001) while the capacities of daily living (IADL) declined from the initial 2.2 to 1.5 (paired *t*-test *p* <  0.001). The proportion of participants showing at least one challenging behaviour increased over 1 year from 76.4 to 79.1% (McNemar *p* <  0.001).

### Mixed-model analyses

Losses to follow-up and persons with missing values in the independent variables were excluded from the linear mixed model, resulting in the analysis of 231 observations. The linear mixed model (Table 3) yielded a significant result (*p* <  0.001, R^2^ = 0.185); however, the only significantly associated factor was the QoL-AD score at baseline. Higher scores at baseline predicted a decline over time (*p* <  0.001; *b* = − 0.357). No other independent variables, including the types of dementia care networks, showed a significant association with the change in QoL-AD scores during the one-year period.

**Table 3:** Linear mixed-model-analyses of quality of life

Dependent variable	*p*-value model	R^2^	independent (Co-) Variables	*p*-value	Beta	95% CI
Proxy-rated quality of life (QoL-AD difference t1-t0)	**< 0.001**	**0.183**				
			**Intercept**	**0.001**	**11.088**	**4.543;17.632**
			Sex: female^a^	0.830	−0.123	−1.220;0.973
			Living situation of caregiver^b^			
			nearby	0.981	−0.017	−1.375;0.134
			greater distance	0.969	0.039	−1.883;1.961
			Social Index (Scheuch-Winkler)^c^			
			middle	0.648	0.291	−0.885;1.467
			high	0.536	0.606	−1.257;2.469
			at least 1 challenging behavior (CMAI)^e^	0.247	0.816	−0.524;2.468
			Type of DCN^d^			
			Organization	0.570	0.632	−1.716;2.980
			Hybrid	0.746	0.301	−1.667;2.269
			Mission	0.509	0.642	−1.401;2.687
			age^f^	0.816	−0.007	−0,069;0.052
			depression (GDS)^f^	0.352	−0.077	−0.233;0.080
			Capacities of daily living (IADL)^f^	0.810	0.039	−0.274;0.352
			Caregiver burden (BIZAD_ADL)^f^	0.118	−0.142	−0.315;0.030
			**QoL-AD at baseline**	**< 0.001**	**−0.357**	**−1.401;-0.231**

^a^ compared to men; ^b^ compared to living together; ^c^ compared to low; ^d^ compared to Stakeholder; ^e^ compared to no challenging behavior. *DCN* Dementia Care Network, ^f^* co-variables, GDS* Geriatric Depression Scale, *CMAI* Cohen-Mansfield-Agitation Inventory, *IADL* Instrumental Activities of Daily Living, *QoL-AD* Quality of Life Alzheimer’s Disease, *BIZAD_ADL* Berliner Inventar zur Angehörigenbelastung - Demenz – Subscale Burden due to practical task

## Discussion

The present paper investigated the quality of life of persons with dementia using different types of dementia care network services, specifically focussing on differences in QoL-AD scores during a one-year follow-up period. In total, 560 participants were recruited, of which 153 persons with dementia with severe impairments in their health-related outcomes dropped out of the study. Generally, the participants of the present study were comparable with those of the IdemUck study [28], in which users of one dementia care network in Germany were studied.

At baseline, there was impairment in IADL-functioning in the users of stakeholder and organisation dementia care networks; consequently, the burden on the primary caregivers was higher in these networks as compared with that in the other two networks. This is to be expected, since higher requirements of (informal) care result in a higher burden. It is evident, using a proxy-rated instrument to assess the quality of life of persons with dementia, that lower capacities of daily living will result in lower quality of life scores [16].

### Quality of life

At baseline, the QoL-AD scores in the present study were comparable with those of the IdemUck study (33, 9) [28]. An in-depth discussion of baseline data has been previously published elsewhere [43]; however, the difference in QoL-AD scores (− 0.4) during the period of one-year is not in accordance with those reported by Vogel et al., [18]. Typically, quality of life declines with the progression of dementia, especially the proxy-rated quality of life. In a sample of community-dwelling persons with dementia, a decline of 2.0 proxy-rated QoL-AD scores was reported by Vogel et al. Since a decrease in quality of life was not observed in the present study, this indicates that using the dementia care network services has a beneficial effect. The analysis of changes over time indicates that quality of life is less stable for users of the stakeholder networks as compared with the other types of dementia care networks. Nonetheless, the multi-level analysis revealed no significant differences among the four types of dementia care networks, suggesting that no type of dementia care network is more beneficial than the others. Further studies should investigate the association of frequency and number of services used.

The only influencing variable predicting the development of quality of life was the baseline QoL-AD score. This is logical, since those with an initial high score will have a greater probability of experiencing a decline in their quality of life scores than people with lower scores at the beginning. Surprisingly, other baseline variables (e.g. depression and IADL functioning) were not significantly associated with changes in quality of life over time, which is in contrast to other previously published studies [16]. It was also unexpected that the living situation of primary caregivers was not significantly associated with this change over time. It is well-known that the frequency of family visits is associated with the quality of life of persons with dementia [45]; thus, it is reasonable that persons living together with their loved ones show higher quality of life scores than others. It is possible that this discrepancy is due to the quality of life being rated by the primary caregiver.

The current results indicate that persons with dementia who were supported by the dementia care network benefited from the support in terms of a steady quality of life over a one-year period. This combination of professional and informal healthcare is a clue to a tailored care provision. This approach, on one hand looks after the needs of a person with dementia, and on the other hand offers support to informal caregivers to decrease their burden [42]. Both aspects are supporting the wishes of persons with dementia to stay at home and potentially delays or even avoids relocations to nursing homes. In Germany, the social healthcare insurance follows the principle of “outpatient rather than inpatient care provision”. The German Federal Ministry of Health sees the benefit of a network of healthcare providers with a special focus on dementia care; therefore in Germany, the governance approved a law that gives financial support to build new dementia care networks and to keep them running. Since 2017, dementia care networks in Germany have been able to apply for grants to support their work.

## Limitations

The present paper comprises several methodological issues that may limit the generalisability of the results. Firstly, with respect to participant selection, although the participating dementia care networks are from all over Germany, the study sample comprises several regional clusters. Additionally, the recruiting process was conducted independently by employees of the dementia care networks, which could influence the representativeness of the study sample. However, the study sample is characteristic and comparable with the samples in other studies; thus, the present findings can be considered valid. Moreover, the benefit of dementia care networks was not clearly evident due to the lack of a control group. Furthermore, health-related outcomes (e.g. FAST) were mostly assessed by informal (non-professional) caregivers as a proxy, and it is possible that professional caregivers may have responded differently, which may have yielded different results.

## Conclusions

To the best of our knowledge, this is the first study to analyse longitudinal results concerning the quality of life of persons with dementia accessing different types of dementia care network services. In general, the presented findings indicate the benefit of dementia care networks as a model of care for community-dwelling persons with dementia. The various governance structures of the regional dementia care networks do not necessarily differentially influence the quality of life of their users. Although dementia care networks can be considered a promising approach for dementia care, further studies are required to investigate the effectiveness of dementia care networks on quality of life, especially in comparison with a control group.

## Abbreviations

ANOVA: Analysis of variance
CMAI: Cohen−Mansfield Agitation Inventory
FAST: Functional Assessment Staging
GDS: Geriatric Depression Scale
IADL: Instrumental Activities of Daily Living
QoL-AD: Quality of Life Alzheimer’s Disease
SD: Standard deviation
